# Evaluation of the efficacy of two doses of vitamin D supplementation on glycemic, lipidemic and oxidative stress biomarkers during pregnancy: a randomized clinical trial

**DOI:** 10.1186/s12884-020-03311-1

**Published:** 2020-10-14

**Authors:** Soudabe Motamed, Bahareh Nikooyeh, Maryam Kashanian, Maryam Chamani, Bruce W. Hollis, Tirang R. Neyestani

**Affiliations:** 1 Department of Cellular and Molecular Nutrition, Shahid Beheshti University of Medical Sciences, Tehran, Iran; 2 Laboratory of Nutrition Research, National Nutrition and Food Technology Research Institute and Faculty of Nutrition Sciences and Food Technology, Shahid Beheshti University of Medical Sciences, Hafezi St., Farahzadi Blvd., Shahrak Qods (Gharb), Tehran, 1981619573 Iran; 3 Department of Obstetrics & Gynecology, Akbarabadi Teaching Hospital, Iran University of Medical Sciences, Tehran, Iran; 4 Division of Neonatology, Department of Pediatrics, Medical University of South Carolina, Charleston, SC 29425 USA

**Keywords:** Vitamin D supplementation, Pregnancy, Glycemic status; lipid profile

## Abstract

**Background:**

Vitamin D deficiency during pregnancy is common and is likely to be associated with metabolic complications in the mother. The aim of this study was to assess the efficacy of two doses of vitamin D supplementation during pregnancy on maternal and cord blood vitamin D status and metabolic and oxidative stress biomarkers.

**Methods:**

The eligible pregnant women (*n* = 84) invited to participate in the study and randomly allocated to one of the two supplementation groups (1000 IU/d vitamin D and 2000 IU/d).

Biochemical assessments of mothers including serum concentrations of 25(OH)D, calcium, phosphate, iPTH, fasting serum sugar (FBS), insulin, triglyceride, total cholesterol, LDL-C, HDL-C, malondialdehyde (MDA) and total antioxidant capacity (TAC) were done at the beginning and 34 weeks of gestation. Cord blood serum concentrations of 25(OH)D, iPTH, MDA and TAC were assessed at delivery as well. To determine the effects of vitamin D supplementation on metabolic markers 1-factor repeated-measures analysis of variance (ANOVA) was used. Between groups comparisons was done by using Independent-samples Student’s *t*-test or Mann-Whitney test. *P* < 0.05 was considered as significant.

**Results:**

Supplementation with 1000 IU/d and 2000 IU/d vitamin D resulted in significant changes in vitamin D status over pregnancy (24.01 ± 21.7, *P* < 0.001 in 1000 IU/d group and 46.7 ± 30.6 nmol/L, *P* < 0.001 in 2000 IU/d group). Daily intake of 2000 compared with 1000 IU/d tended to increase the serum concentration of HDL-C (10 ± 8.37, *P* < 0.001 in 1000 IU/d group and 9.52 ± 11.39 mg/dL, *P* < 0.001 in 2000 IU/d group). A significant decrement in serum concentration of iPTH observed in both groups (− 4.18 ± 7.5, *P* = 0.002 in 1000 IU/d group and − 8.36 ± 14.17, *P* = 0.002 in 2000 IU/d group).

**Conclusions:**

Supplementation with 2000 IU/d vitamin D as compared with 1000 IU/d, is more effective in promoting vitamin D status and HDL-C serum concentration and in decreasing iPTH over pregnancy.

**Trial registration:**

This trial is registered at clinicaltrials.gov (NCT03308487). Registered 12 October 2017 ‘retrospectively registered’.

## Background

Physiologic alterations during pregnancy cause changes in the concentrations of many circulating metabolites and analytes including glucose, lipids and lipoproteins. The extent of these changes highly depends on interactions of dietary intake, genetic makeup and hormonal milieu [1–3].

Among the modifiable factors affecting gestational blood glucose and lipid alterations, nutrition has a crucial role. Apart from proper gestational weight gain and adhering to a healthy diet prior to and throughout pregnancy, vitamin D status of mother has attracted a huge attention for some reasons [4, 5]. Several research groups have scrutinized the relationship between vitamin D deficiency and abnormal glucose homeostasis [6–10] and lipid metabolism [11] in pregnancy. Furthermore, some clinical trials have revealed that vitamin D supplementation during pregnancy might improve the status of lipid profile [12–14], glycemia [15], and parathyroid hormone [16].

It is believed that certain level of oxidative stress (OS), both placental and maternal, is necessary for a normal pregnancy [17, 18]. However, pathological increment of OS during pregnancy could have a role in development of adverse pregnancy outcomes through damaging susceptible placenta [19, 20]. On the other hand, some studies have revealed antioxidative properties of vitamin D [21–23] which might be associated with certain metabolic variables, notably blood glucose and lipids [24]. However, these effects of vitamin D during pregnancy still need further elucidation.

High prevalence of vitamin D deficiency during pregnancy is common in most countries of the world [25, 26], including Iran [27, 28]. It is noteworthy that the Iran Ministry of Health (IrMOH) communicated with all governmental health centers to prescribe 1000 IU/d vitamin D supplement to all pregnant women from the beginning of pregnancy. However, the efficacy of supplementation with this dosage (1000 IU/d) early in pregnancy has not yet been evaluated and compared with that of 2000 IU/d, which has been more efficient in improving vitamin D status and depressing certain inflammatory biomarkers, than 1000 IU/d [29]. We, therefore, designed this clinical trial from the first trimester of pregnancy to examine and compare the efficacy of these two dosages of vitamin D (1000 IU/d and 2000 IU/d) on certain metabolic parameters including glycemic, lipidemic and parathyroid hormone as well as OS status.

## Methods

### Study design

This study was a part of a larger project whose complete protocol has been comprehensively described elsewhere [30]. In an open-label randomized clinical trial, 84 pregnant women who met the inclusion criteria and were attending the outpatient obstetric clinics of three hospitals (with similar cultural, educational and economic status) in Tehran between February 2017 and January 2018 were enrolled. This study adhered to CONSORT guidelines.

We calculated that a sample of 37 subjects in each group would have 90% power to detect a change in means of 25(OH)D of 0.75 of SD (assuming an effect size of 0.75). Based on the previous study [31], 0.75 of SD would be 18 nmol/L.

Inclusion criteria were: 1) being at the first trimester of pregnancy, 2) the absence of any clinical disease including endocrine, cardiovascular, liver and kidney diseases, 3) not taking vitamin D (> 600 IU/d) and/or omega-3 supplements and/or steroids during the past 3 months, 4) the willingness to take part in the study.

Exclusion criteria were: 1) identified to have FBS > 92 mg/dL or blood pressure > 140/90 mmHg at the first visit, 2) consuming omega-3 and/or extra vitamin D supplement and/or other drugs that interfere with vitamin D metabolism during the intervention period, 3) poor compliance to the supplementation 4) unwillingness to continue the intervention.

Those subjects who met the inclusion criteria were randomly allocated to one of the two groups to take either 1000 IU (one tablet)/d or 2000 IU (two tablets)/d vitamin D from the first trimester till the end of pregnancy. Block randomization applied using 6 blocked sizes of 4 to generate 21 randomized block allocations for random allocation. All participants were allowed to receive the common supplementation during pregnancy (folic acid, iron and multivitamins providing < 600 IU/d vitamin D). The vitamin D3 tablets were purchased from Jalinous pharmaceutical company, Tehran, Iran.

All subjects were visited at first trimester and 34–36 week of gestation. At the first visit, demographic information was collected from all participants. Adherence to the determined supplementation regimen was evaluated as described earlier [30]. Briefly, the participants were asked to return the pills not consumed for any reason. Meanwhile, they were contacted by telephone call every week to check consumption of the supplements. The following equation was applied to assess the adherence rate: (Number of pills dispensed − number of pills remained)/(prescribed number of pills per day × number of days between 2 visits. Accordingly, the participants divided into three groups including strictly (> = 80%), moderately (50–80%) and poorly (≤50%) compliant. Those participants with poor compliance (consumption of less than 50% of the prescribed vitamin D supplements in a month) were excluded from the study. The strictly and moderately compliant subgroups supposed to be separately analyzed.

Anthropometric and blood pressure measurements have been described elsewhere [30].

To determine the mean score of sunlight exposure, the information about the amount of time spent outdoors each day during previous week were collected by using the weekly sunlight exposure recall questionnaire [32]. Then the score of time outdoors (score 0 for ≤5 min; score 1 for 5–30 min; score 2 for ≥30 min) and the score of the amount of skin exposure (score 1 for face and hands only; score 2 for face, hands, and arms; score 3 for face, hands, and legs; score 4 for almost the entire body) were multiplied to calculate sunlight exposure score. The sunlight exposure score range is assumed to be 0 to 56 [32].

To assess the level of physical activity, an Iranian version of the International Physical Activity Questionnaire (IPAQ) was used [33].

A 24-h recall questionnaire was used to assess the amount of energy, macronutrient (protein, carbohydrate and fat) and micronutrient intake (vitamin A, D, E, K, C, calcium, iron, zinc) for 2 days (a week day and a weekend day), the mean of which was considered as the individual’s dietary intake. The analysis of questionnaires data was done using Nutritionist IV software (First Databank, San Bruno, CA, USA) modified for Iranian foods.

The protocol of this study was approved by the Ethics Committee of Shahid Beheshti University of Medical Sciences. This trial is registered at clinicaltrials.gov (NCT03308487).

### Biochemical investigations

Ten mL of 12–14 h fasting venous blood and spot urine samples were collected from all participants at first trimester and 34–36 weeks of gestation. In addition, 5 mL of cord blood samples were collected at the time of delivery. All blood and urine samples were transferred to the Laboratory of Nutrition Research, NNFTRI, in a cold box. Blood samples were centrifuged in less than 2 h at 800 *g* at room temperature for 30 min. The separated sera and urine samples were aliquoted and kept at − 80 °C freezer for further analyses. Sera from cord blood samples were handled similarly.

Fasting serum glucose, lipid profile components, calcium and phosphorous were assayed on the same day of blood sampling using commercial kits (all from Pars-Azmoon, Tehran, Iran) and an auto-analyzer (Selecta E; Vitalab, Holliston, Netherlands).

Fasting serum insulin was measured by using an enzyme immunoassay (EIA) kit (DiaPlus inc., Canada) with intra- and inter-assay coefficient of variations (CVs) of 4.9 and 8%, respectively, based on manufacturer’s data.

HOMA-IR which calculated based on suggested formulas [34], was used to assess insulin resistance.

The concentrations of 25(OH)D and iPTH in all serum samples were determined using commercial enzyme-immunoassay (EIA) kits (both from EUROIMMUN, Leubeck, Germany). For 25(OH)D assay kit, the inter-assay CVs ranged from 7 to 8.6- and intra-assay CVs ranged from 3.2 to 6.9% whereas for iPTH intra- and inter-assay CVs were 2.2–9.5% and 9.5–11%, respectively, according to the manufacturer.

Urinary calcium and creatinine concentrations were measured by commercial kits and an auto-analyzer. The proportion of urinary calcium to creatinine concentrations in the spot urine samples was used to determine the possible effects of vitamin D supplementation on urinary calcium excretion.

To evaluate OS, serum concentrations of malondialdehyde (MDA) as well as total antioxidant capacity were determined. MDA assay was performed using thiobarbituric acid reacting substances (TBARS), as previously described [29]. To determine total antioxidant capacity (TAC), the ability of serum antioxidants to inhibit 2,2ˊ-azino di-(3-ethylbenzthiazoline sulfonate; ABTS) oxidation was compared with that of bovine serum albumin, as the standard [35].

### Statistical analysis

To check normal distribution of continuous variables the histogram and Kolmogorov-Smirnov test were applied.

The continuous data which normally distributed shown as mean and standard deviation (SD) and categorical data presented as frequency or percentage (%). Repeated-measures ANOVA was used to determine the effect of vitamin D supplementation on metabolic markers. In this analysis, the treatment (2000 IU/d vitamin D vs. 1000 IU/d vitamin D) and time (with 2 time points including baseline and week 34 of the intervention) were considered as “between-subject factor” and “within-subject factor” respectively. For between groups comparison, Independent-samples Student’s *t*-test or Mann-Whitney test was used. Either Pearson (r) or Spearman (r_s_) equations were applied to assess the correlations between variables. The significant level was *P* < 0.05.

## Results

**Fig. 1:**
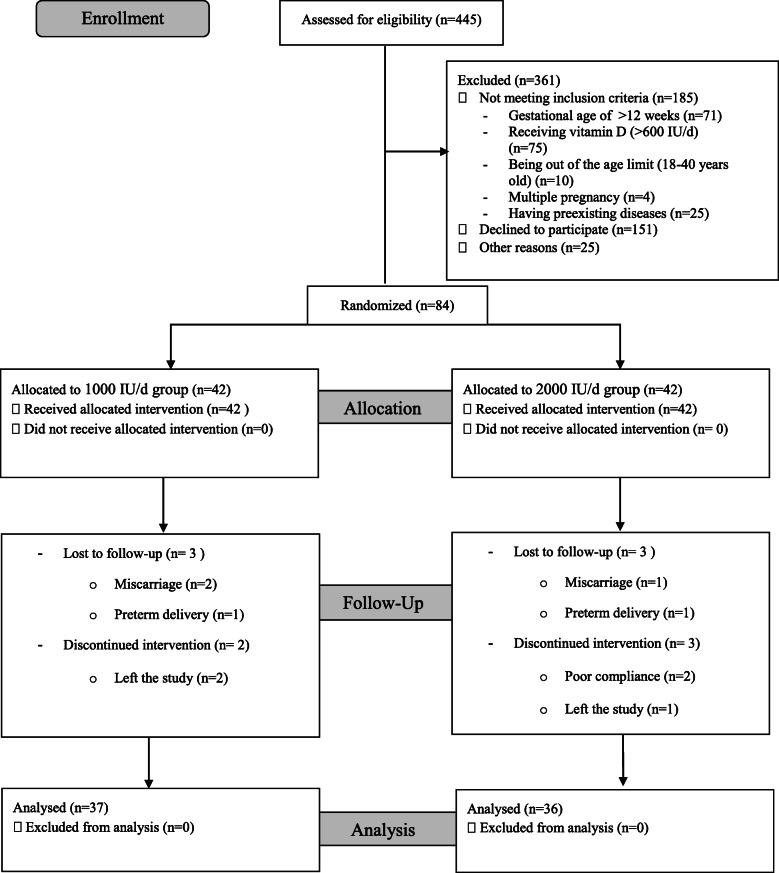
Flow diagram of the study

A total of 84 pregnant women were enrolled in the study between February and June 2017. However, 73 subjects (*n* = 37 from group 1000 IU/d and *n* = 36 from group 2000 IU/d) completed the intervention. Losses and exclusions were due to some reasons including miscarriage, preterm delivery, and unwillingness to continue the participation (Fig. 1). Follow up period lasted till January 2018.

In this study 2 subjects (about 5%) from 2000 IU/d group had poor compliance and were excluded from the study. The mean adherence percent among those who continued the study were 93.7 and 90.4% in 1000 IU/d and 2000 IU/d groups respectively. It is necessary to mention that all participants who completed the study were belonged to the strictly compliant subgroup. Therefore, the study was out of moderately compliant subgroup.

The baseline characteristics, sun exposure score and physical activity level of the participants, duration of intervention, number (percent) of participants who have received routine supplementation and season of year at the first visit have been shown in Table 1.

**Table 1:** Baseline characteristics of subjects^a^

Characteristic	Group 1 (1000 IU/d) n_1_ = 37	Group 2 (2000 IU/d) n_2_ = 36	*p* value ^2^
**Age**, year	27.94 ± 5.60	27.41 ± 5.15	0.80
**Gestational age at enrollment**, week	10.39 ± 1.69	10.08 ± 2.17	0.77
**Gestational age at delivery** (week)	38.79 ± 1.65	39.91 ± 5.78	0.57
**Weight gain at end of pregnancy** (kg)	10.82 ± 5.21	12.01 ± 5.94	0.38
**Weekly sunlight exposure score at enrollment**	2.63 ± 2.08	1.97 ± 1.67	0.14
**Weekly sunlight exposure score at last visit**	1.70 ± 1.65	1.21 ± 1.15	0.17
**Sunscreen use**, n (%)	11 (30.4)	13 (36.9)	0.49
**Physical activity at enrollment**, n (%)			0.37
Low	29 (78.4)	28 (77.8)	
Moderate	8 (21.6)	6 (16.7(	
High	0 (0.0)	2 (5.6)	
**Physical activity at last visit**, n (%)			0.59
Low	32 (86.5)	29 (80.6)	
Moderate	5 (13.5)	6 (16.7)	
High	0 (0.0)	1 (2.8)	
**Duration of intervention**, n (%)			0.87
24 wk	11 (29.72)	10 (27.77)	
25 wk	9 (24.32)	11 (30.55)	
26 wk	6 (16.21)	7 (19.44)	
27 wk	7 (18.91)	5 (13.88)	
28 wk	4 (10.81)	3 (8.33)	
**Season at first visit**, n (%)			0.89
Winter	14 (37.6)	16 (44.4)	
Spring	23 (62.1)	20 (55.5)	
**Routine supplementation**^a^, n (%)			
Multivitamin	25 (67.5)	22 (61.1)	0.52
Folic acid	29 (78.37)	25 (69.44)	0.37
Iron	21 (56.75)	22 (61.11)	0.55

^a^data are expressed as mean ± SD and the distribution between groups expressed as n (%)

^2^t test and χ2 test were used to compare baseline characteristics

*BMI* Body mass index

^a^The participants started to intake routine supplementation late in the fourth month of pregnancy

Vitamin D deficiency/insufficiency was found in 83.8 and 86.2% of the subjects in 1000 IU/d group and 2000 IU/d groups, respectively (Table 3).

The increment of the 25(OH)D was significantly higher in response to vitamin D supplementation with the dose of 2000 IU/d compared with 1000 IU/d (Tables 2 and 3). No significant between groups difference was found in the cord blood concentration of 25(OH)D (Table 4). The cord blood serum concentrations of 25(OH) D had a significant positive correlation with the maternal one at third trimester (*r* = 0.38, *P* = 0.02).

**Table 2:** Comparison of changes in variables within and between groups after the intervention^a^

Group Variable	1000 IU/d group *n* = 37			2000 IU/d group *n* = 36			*p* value^2^		
	before	after	changes	before	after	changes	Time	Group	Time × group^b^
**25(OH)D3** (nmol/ liter)	45.32 ± 29.70	71.19 ± 23.65*	24.01 ± 21.7	47.03 ± 31.70	91.82 ± 28.81*	46.7 ± 30.6 **	< 0.001	0.06	0.004
**FBS** (mg/dl)	74.70 ± 7.51	75.59 ± 6.98	0.89 ± 11.06	78.41	77.55	−0.86 ± 10.91	0.99	0.054	0.49
**Insulin** (mU/L)	7.97 ± 4.85	8.82 ± 5.47	0.85 ± 6.15	7.95 ± 5.96	10.40 ± 9.31	2.44 ± 10.28	0.11	0.53	0.44
**HOMA_IR**	1.45 ± 0.85	1.52 ± 1.21	0.21 ± 1.23	1.66 ± 1.11	2.03 ± 1.84	0.50 ± 2.03	0.08	0.37	0.47
**TG** (mg/dl)	98.59 ± 34.10	245.43 ± 68.75*	146.83 ± 59.16	97.22 ± 49.94	228.41 ± 75.03*	131.19 ± 58.16	< 0.001	0.44	0.26
**TC** (mg/dl)	153.78 ± 29.05	229.83 ± 48.84*	76.05 ± 38.110	158.22 ± 36.58	228.44 ± 32.13*	70.22 ± 35	< 0.001	0.49	0.84
**HDL** (mg/dl)	52.08 ± 8.68	62.08 ± 10.61*	10 ± 8.37	54.69 ± 12.86	64.22 ± 15.78*	9.52 ± 11.39	< 0.001	0.36	0.84
**LDL** (mg/dl)	81.98 ± 20.93	118.67 ± 37.38*	36.68 ± 29.93	84.08 ± 26.06	118.53 ± 23.89*	34.45 ± 27.39	< 0.001	0.86	0.74
**LDL/HDL-C**	1.58 ± 0.36	1.90 ± 0.44*	0.31 ± 0.44	1.56 ± 0.45	1.91 ± 0.46*	0.35 ± 0.47	< 0.001	0.92	0.76
**TC/HDL-C**	2.97 ± 0.44	3.71 ± 0.49*	0.73 ± 0.51	2.93 ± 0.55	3.67 ± 0.63*	0.73 ± 0.52	< 0.001	0.68	0.99
**Calcium** (mg/dl)	9.47 ± 0.38	8.97 ± 0.37*	−0.5 ± 0.41	9.45 ± 0.39	9.01 ± 0.32*	−0.43 ± 0.45	< 0.001	0.88	0.51
**Phosphate** (mg/dl)	4.37 ± 0.45	4.63 ± 1.0	0.26 ± 1.06	4.45 ± 0.62	4.45 ± 0.49	0.0 ± 0.69	0.21	0.67	0.21
**iPTH** (pg/ml)	17.96 ± 10.60	13.78 ± 7.05*	−4.18 ± 7.5	23.51 ± 15.77	15.15 ± 7.68*	−8.36 ± 14.17	< 0.001	0.11	0.13
**MDA** (nmol/mL)	21.17 ± 8.13	17.6 ± 8.7	−1.64 ± 10.42	18.87 ± 8.37	18.17 ± 8.07	1.9 ± 8.36	0.52	0.46	0.26
**TAC** (g/dL BSA equivalent)	2.99 ± 0.37	2.79 ± 0.58	−0.19 ± 0.67	2.84 ± 0.55	2.78 ± 0.45	−0.06 ± 0.62	0.18	0.51	0.50

^a^All values are expressed as means±SD (standard deviation)

^2^Resulted from 1-factor repeated measures ANOVA

*different from baseline, *p* < 0.05

**difference of changes between groups, *P*-interaction< 0.05

^b^Time×Group: Time means from the first to the last visit of intervention and group means comparison between the two groups of intervention

*25(OH)D3* 25-hydroxyvitamin D3, *FBS* fasting serum glucose, *TC* total cholesterol, *TG* triglycerides, *LDL* low-density lipoprotein, *HDL* high-density lipoprotein, *iPTH* intact parathyroid hormone, *MDA* malondialdehyde, *TAC* total antioxidant capacity, *HOMA-IR* homeostasis model assessment of insulin resistance

**Table 3:** Comparison of vitamin D status based on serum concentration of 25(OH)D between groups

Group	Before intervention			After intervention			*p* value^1^
	*Deficient*	*Insufficient*	*Sufficient*	*Deficient*	*Insufficient*	*Sufficient*	
**1000 IU/day**, n (%)	26 (70.3)	5 (13.5)	6 (16.2)	3 (8.1)	20 (54.1)	14 (37.8)	0.04
**2000 IU/day**, n (%)	20 (55.6)	11 (30.6)	5 (13.9)	2 (5.6)	10 (27.8)	24 (66.7)	
**Total**, n (%)	46 (63)	16 (21.9)	11 (15.1)	5 (6.8)	30 (41.1)	38 (52.1)	

^1^Denotes the significance of differences in the distribution of vitamin D categories between the 2 groups (chi-square test)

Deficiency is defined as < 50 nmol/L, insufficiency as 50–75 nmol/L, and sufficiency as > 75 nmol/L

**Table 4:** Cord blood biomarkers^a^

Group	1000 IU/d Group *n* = 15	2000 IU/d Group *n* = 18	*p* value^2^
Variable			
**25 (OH)D** (nmol/L)	84.04 ± 35.14	89.0 ± 25.27	0.64
**Cord blood 25(OH)D**, n (%)			0.23
< 50 nmol/l	3 (20)	0 (0.0)	
50–75 nmol/l	4 (26.7)	7 (38.9)	
> 75 nmol/l	8 (53.3)	11 (61.1)	
**iPTH** (pg/ml)	4.53 ± 2.82	4.70 ± 2.95	0.87
**MDA** (nmol/mL)	20.33 ± 5.9	20.55 ± 9.43	0.91
**TAC** (g/dL BSA equivalent)	3.15 ± 0.97	3.36 ± 0.91	0.58

^a^All values are expressed as means (SD, standard deviation)

^2^Denotes the significance between groups differences (independent t-test)

*25(OH)D3* 25-hydroxyvitamin D3, *iPTH* intact parathyroid hormone, *MDA* malondialdehyde, *TAC* total antioxidant capacity

Within- or between-group changes of FBS, serum insulin, HOMA-IR were not significant. Compared with the baseline values the concentration of TC, TG, LDL-C and HDL-C increased significantly in both groups. However, between group changes were not significant (Table 2). A small but statistically significant decrease in serum calcium concentrations at late pregnancy was observed in both groups (*P* < 0.001), although the values remained in normal range. However the between-group difference was not significant (*P*-interaction = 0.51). Serum phosphate concentration did not differ over pregnancy in both groups (*P*-interaction = 0.21).

Final serum concentrations of iPTH in both groups decreased late in pregnancy (*P* < 0.05) but the difference between groups was not significant (Table 2). The cord blood concentration of iPTH was not significantly different between groups, either (Table 4). A significant negative correlation was found between serum concentrations of 25(OH)D and iPTH (*r* = − 0.35, *P* = 0.003). The correlation between mothers’ and cord blood serum concentration of iPTH was not significant (*r* = 0.19, *P* = 0.32).

The biomarkers of OS (MDA and TAC) did not change significantly over pregnancy in any of the groups (Table 2). Accordingly, cord blood MDA and TAC did not show any significant between-group changes, either (Table 4).

Initial and final dietary data showed no significant between group differences (Table 5).

**Table 5:** Comparison of changes in energy and nutrient intake within and between groups after the intervention^a^

Group	DRIs^c^	1000 IU/d group *n* = 37			2000 IU/d group *n* = 36			*p* value^2^		
Variable		before	after	changes	before	after	changes	Time	Group	Time × group^b^
**Energy** (kcal/d)	**	1792.2 ± 280.1	1892.9 ± 396.8	100.7 ± 88.78	1675.1 ± 416.3	1776.19 ± 536.58	118.32 ± 136.24	0.2	0.59	0.34
**Protein** (g/d)	71	70.6 ± 27	77.43 ± 20.7	6.76 ± 6.68	64.76 ± 27.31	63.05 ± 53	−1.29 ± 6.77	0.28	0.47	0.94
**Carbohydrate** (g/d)	175	266.81 ± 83.62	277.56 ± 72.54	10.74 ± 15.86	261.56 ± 71.08	275.63 ± 104.32	17.05 ± 26.51	0.27	0.38	0.33
**Fat** (g/d)	***	51.69 ± 26.23	55.8 ± 16.73	4.1 ± 5.14	43.96 ± 15.06	49.84 ± 20.14	6.28 ± 4.88	0.19	0.11	0.65
**Calcium** (mg/d)	1000	663.33 ± 268.9	771.67 ± 439.32	108.34 ± 72.93	593.46 ± 235.82	554.97 ± 238.3	−38.48 ± 66.06	0.29	0.006	0.17
**Iron** (mg/d)	27	12.56 ± 4.59	13.61 ± 5.93	1.04 ± 1.35	12.14 ± 4.42	15.73 ± 14.21	1.07 ± 1.09	0.14	0.65	0.41
**Zinc** (mg/d)	11	7.44 ± 4.13	8.03 ± 2.99	0.58 ± 1.03	6.29 ± 3.25	6.71 ± 3.29	0.37 ± 0.94	0.25	0.39	0.64
**Vitamin A** (RE/d)	770	1234.8 ± 1549.1	1097.6 ± 1958.5	− 137.1 ± 20.1	884.41 ± 713.8	1006.82 ± 940.03	217.4 ± 215.9	0.87	0.059	0.47
**Vitamin D** (μg)	15	0.7 ± 1.01	1.27 ± 1.45	0.57 ± 0.32	0.45 ± 1.00	0.90 ± 1.34	0.37 ± 0.36	0.03	0.23	0.81
**Vitamin E** (α-TE (mg/d))	15	3.13 ± 2.52	4.78 ± 7.11	1.65 ± 1.54	3.71 ± 2.85	4.74 ± 5.25	1.15 ± 1.27	0.17	0.78	0.75
**Vitamin C** (mg/d)	85	135.01 ± 116.3	84.93 ± 46.87	−50.08 ± 26.7	86.02 ± 52.14	145.55 ± 100.48	4.03 ± 21.2	0.88	0.86	0.10

^a^All values are expressed as means±SD (standard deviation)

^2^Resulted from 1-factor repeated measures ANOVA

^b^Time×Group: Time means from the first to the last visit of intervention and group means comparison between the two groups of intervention

^c^*DRIs* Dietary Reference Intakes [36]

**Energy requirement varies widely based on the pre-pregnancy BMI, individual energy output and basal metabolic rate

***There is no DRI for lipids during pregnancy. It is based on the energy requirements for proper weight gain

## Discussion

We found a relatively high proportion of vitamin D deficiency and insufficiency among study participants at first trimester of pregnancy. This finding is consistent with what has been reported by several studies about the percentage of vitamin D deficiency among Iranian pregnant women [27].

In addition, taking 2000 IU/d vitamin D_3_ for 24 weeks improved vitamin D status more effectively than taking 1000 IU/d. This finding was compatible with that of other trials examined the efficacy of similar doses [37–39].

Sufficient concentrations of cord blood 25(OH)D (> 75 nmol/L) were observed in both groups with no significant between-group difference. Along the same line of evidence, the daily intake of 2000–4000 IU vitamin D_3_ among women from 12 to 16- weeks of gestation until delivery prevented neonatal vitamin D deficiency [40].

Our study showed no association between vitamin D supplementation and parameters of glucose homeostasis including FBS, insulin and HOMA-IR during pregnancy. In agreement with our findings, some studies failed to show any beneficial effects of vitamin D supplementation on glycemic status of pregnant women [7, 8, 41, 42]. The results of some cohort studies showed that the concentrations of circulating 25(OH)D might not be a contributing factor for development of GDM in women with a low risk for GDM [9, 43]. On the contrary, the results of a clinical trial showed that co-administration of 1000 mg calcium per day + 50,000 IU vitamin D every 3 weeks for 6 weeks to pregnant women with GDM could improve glycemic status [44]. The effect of vitamin D supplementation on glycemic status has been attributed to its involvement in insulin secretion through its effect on the regulation of serum calcium which in turn affects pancreatic β cell function [45]. The observed inconsistency might be due to the differences in doses and duration of vitamin D supplementation and the lack of calcium co-supplementation and the fact that in our study, vitamin D_3_ was administered to healthy pregnant women with normal values of glycemic status.

We found a significant increase in serum concentrations of lipid profile components including TG, total cholesterol, HDL-C and LDL-C from beginning to the late pregnancy in both groups, but the differences were not significant between groups. During pregnancy along with increasing gestational age the levels of lipid profile increase most probably due to hormonal and metabolic changes as a normal physiologic phenomena [46–49]. The normal ranges of TG, TC, LDL-C, HDL-C during third trimester of pregnancy suggested to be 131–453, 219–349, 101–224 and 48–87 mg/dL respectively [50–52]. Nevertheless, the importance of changes of blood lipid metabolism during pregnancy pertains to its potential effect on perinatal morbidity and mortality. It has been reported that dyslipidemia in pregnancy associated with preeclampsia, GDM, preterm delivery and cardiovascular disease in the future [47]. Based on some evidence, vitamin D deficiency during early pregnancy associated with more unfavorable changes of lipid profile [53, 54]. Therefore, it is assumed that vitamin D correction could have a beneficial effect on lipids during pregnancy. Asemi, et al. conducted a randomized, double-blind, placebo-controlled clinical trial in 57 women with GDM that received 50,000 IU vitamin D at baseline and at day 21 of the intervention and those in the placebo group received 2 placebos at the same times. Based on the results, vitamin D supplementation resulted in increased serum 25-hydroxyvitamin D concentrations compared with placebo and a significant reduction in concentrations of total cholesterol and LDL-C [55]. In another study, supplementation of vitamin D among GDM cases showed no positive effect on lipid profiles compared with placebo. Although, serum concentration of total cholesterol and LDL-C had a significant increment in the placebo group [56]. Inconsistently, the results of a trial among pregnant women, who were prone to pre-eclampsia, revealed that supplementation of 50,000 IU vitamin D, every 2 weeks from gestational age of 20 to 32 weeks, in comparison to placebo, associated with increased level of HDL-C concentrations [57]. The possible effect of vitamin D on lipid metabolism could be in part explained by decreased synthesis and secretion of TG in liver and also transformation of cholesterol into bile acids and subsequently reduced level of cholesterol [58].

In the present study due to the lack of control group we were unable to detect the effect of vitamin D supplementation on the lipid profile. However, we found that the effect of vitamin D supplementation with two doses of 1000 IU/d and 2000 IU/d on the lipid profile during pregnancy were not different. Future studies with higher and safe doses of vitamin D supplementation may provide better explanations. The present study detected a significant reduction in maternal serum iPTH concentration in both groups at the end of pregnancy, and a negative correlation between serum concentrations of 25(OH)D and iPTH. In agreement with our study results, an interventional study of three treatment groups (400, 2000 or 4000 IU vitamin D3/day) from 12 weeks of gestation until delivery, showed that the circulating PTH of African American group had inverse correlation with circulating 25(OH)D levels [59]. Okonofua et al., found an inverse correlation between serum 25(OH)D and PTH concentrations in the maternal serum but the inverse relationship disappeared when the ethnicity of women was considered separately [60].

We found no correlation between mothers’ sera and cord blood concentrations of iPTH or cord blood concentrations of 25(OH)D and iPTH. Similar to our results, Bowyer et al. showed no correlation between PTH in neonates and their mothers [61]. Considering the importance of minerals for fetus bone development, active transport of calcium occurs across the placenta which subsequently suppresses PTH secretion [62]. Hirota et al. have shown that there is a significantly higher level of the parathyroid hormone-related protein (PTHrp) in umbilical venous blood than those in pregnant women at any trimester [63]. PTHrp has an important role in active transferring of calcium from mother to the fetus, possibly via changing the expression of calbindin-D 9 k [64].

We found no significant changes in maternal serum concentration of MDA and TAC at the end of intervention in any of the two groups and also no significant between-group difference of these biomarkers in cord blood. Similar to our data the findings of a trial showed that vitamin D supplementation had no effect on OS biomarkers among pregnant women with GDM [55]. However, there are some evidence that support a positive association between TAC and vitamin D status [41, 65]. The findings of a systematic review of clinical trials among non-pregnant participants revealed that vitamin D only with doses of 100,000–200,000 IU/month could have beneficial effect on the OS parameters (decreases MDA levels and increases GSH and TAC levels) [23]. One of these mechanisms that explain the effect of vitamin D on OS biomarkers is the role of vitamin D on the expression of Nrf2 which in turn increases the production of anti-oxidant enzymes [66]. The lack of changes in OS biomarkers in the present study might, at least in part, be related to the applied doses of vitamin D. Therefore, further studies with different doses could clarify the role of vitamin D supplementation on the OS biomarkers in pregnant women.

One of the strength points of this randomized clinical trial is that vitamin D supplementation was started early in pregnancy and lasted until delivery. We also assessed dietary intake, physical activity level and sun exposure to find their possible confounding effects. Biochemical markers were measured in cord blood samples for better understanding of the effects of the intervention. Nonetheless, some limitations must be acknowledged. We did not have a placebo control group (no vitamin D intake via supplements including multivitamin) due to ethical issues. Yet, our participants were normo-glycemic and normo-lipidemic and this might hamper the possible effects of the intervention. However, this precaution was necessary as this trial was performed to help policy-makers at the Iran Ministry of Health to decide on vitamin D supplementation during pregnancy for the whole country.

## Conclusions

Supplementation with 2000 IU/d vitamin D had no more beneficial effects on the studied biomarkers of glycemic, lipidemic and OS status of the maternal and cord blood than with 1000 IU/d. Nevertheless, supplementation with 2000 IU a day, compared with 1000 IU/d, was more effective in improving vitamin D status and lowering the occurrence of suboptimal circulating calcidiol concentrations during pregnancy.

## Data Availability

The datasets used and/or analyzed during the current study are available from the corresponding author on reasonable request.

## Abbreviations

ABTS: 2,2ˊ-azino di-(3-ethylbenzthiazoline sulfonate)
Ca: Calcium
CV: Coefficient of variation
FBS: Fasting blood sugar
GDM: Gestational diabetes mellitus
HDL-C: High-density lipoprotein-cholesterol
HOMA-IR: Homeostatic model assessment of insulin resistance
25 (OH) D: 25-hydroxycalciferol
IOM: Institute of Medicine
iPTH: Intact parathyroid hormone
LDL-C: Low-density lipoprotein cholesterol
MDA: Malondialdehyde
OS: Oxidative stress
SD: Standard deviation
SEM: Standard error of mean
TAC: Total antioxidant capacity
TC: Total cholesterol
TG: Triglyceride
